# Endothelium-Independent Hypoxic Contraction Is Prevented Specifically by Nitroglycerin via Inhibition of Akt Kinase in Porcine Coronary Artery

**DOI:** 10.1155/2016/2916017

**Published:** 2015-12-29

**Authors:** Huixia Liu, Yanjing Li, Yuanming An, Peixin He, Liling Wu, Yuansheng Gao, Dou Dou

**Affiliations:** ^1^Department of Physiology and Pathophysiology, Peking University Health Science Center, 38 Xue Yuan Road, Beijing 100191, China; ^2^Department of Physiology, Heze Medical College, Heze, Shandong, China; ^3^Key Laboratory of Molecular Cardiovascular Science (Peking University), Ministry of Education, Beijing, China

## Abstract

* Objective*. Hypoxia-induced sustained contraction of porcine coronary artery is endothelium-independent and mediated by PI3K/Akt/Rho kinase. Nitroglycerin (NTG) is a vasodilator used to treat angina pectoris and acute heart failure. The present study was to determine the role of NTG in hypoxia-induced endothelium-independent contraction and the underlying mechanism.* Methods and Results*. Organ chamber technique was used to measure the isometric vessel tension of isolated porcine coronary arteries. Protein levels of phosphorylated and total Akt were determined by western blot. A sustained contraction of porcine coronary arteries induced by hypoxia was significantly reduced by NTG but not by isoproterenol. This contraction was also inhibited by DETA NONOate, 8-Br-cGMP, which can be reversed by ODQ, and Rp-8-Br-PET-cGMPS. The restored contraction was blocked by LY294002. The reduction of Akt-p at Ser-473 by NTG, DETA NONOate, and 8-Br-cGMP was significantly inhibited by ODQ, PKG-I. The decrease in Akt-p level by NTG and 8-Br-cGMP was prevented by calyculin A but not by okadaic acid.* Conclusions*. These results demonstrated that the endothelium-independent sustained hypoxic vasoconstriction can be prevented by NTG and that the inhibition of PI3K/Akt signaling pathway may be involved.

## 1. Introduction

Since it was firstly reported by Vanhoutte in 1976 that acute hypoxia caused a rapid increase in tension of canine saphenous veins precontracted with acetylcholine [1], hypoxic vasoconstriction has been observed in a number of systemic vessel types including coronary arteries [2–9]. Subsequent studies demonstrate that the rapid hypoxic contraction is endothelium-dependent and closely associated with nitric oxide (NO) and soluble guanylyl cyclase (sGC) but unrelated to cGMP [4, 7, 8]. Our recent study revealed that cIMP, which was formed by sGC under hypoxic conditions, may play a role in the hypoxia-induced vasoconstriction [9]. This rapid endothelium-dependent hypoxic vasoconstriction is usually followed by a transient relaxation and then a sustained contraction in porcine coronary arteries when exposed to hypoxia. Our recent study suggests that this sustained hypoxic contraction is endothelium-independent and mediated by PI3K/Akt/Rho kinase [10].

Akt is a serine/threonine kinase which plays important roles in cell survival, differentiation, proliferation, metabolism, migration, and apoptosis [11–16]. It is increasingly recognized as being a pivotal modulator of vascular tone by regulating endothelial nitric oxide synthase, L-type calcium channel, Rho kinase, and phosphodiesterase type 5 [17–25]. Activation of Akt depends on the integrity of its pleckstrin homology (PH) domain, which is required for the membrane translocation of the kinase. After being translocated to the membrane via binding of phosphatidylinositol (3,4,5)-triphosphates, Akt is phosphorylated by its upstream kinases at Thr-308 and then at Ser-473 [26]. Akt is not fully activated until both Thr-308 and Ser-473 are phosphorylated. Therefore, the full activation of Akt depends on the phosphorylation of Ser-473 [27]. Our recent study showed that the hypoxia-induced Akt phosphorylation at Ser-473 is associated with changes in the tension of porcine coronary arteries [10].

Nitroglycerin (NTG) is a commonly used medicine in the treatment of angina pectoris and acute heart failure. It causes vasodilatation after being converted into NO or a NO related intermediate in the cytoplasm, followed by cGMP elevation due to activation of sGC and PKG [28, 29]. Our recent study showed that Akt activity was inhibited by NO via inhibiting the phosphorylation at Ser-473 of the enzyme in porcine pulmonary artery [30]. The present study was intended to determine whether the endothelium-independent hypoxic contraction is prevented by NTG via inhibition of Akt in porcine coronary artery.

## 2. Materials and Methods

### 2.1. Reagents

The following drugs were used (unless otherwise specified, all were obtained from Sigma, St. Louis, MO, USA): 8-Br-cGMP (8-bromo-guanosine 3′5′-cyclic monophosphate), calyculin A, DETA NONOate[2,2′-(hydroxynitrosohydrazono) bis(ethanamine)], LY294002 [2-(4-morpholinyl)-8-phenyl-1(4H)-benzopyran-4-one hydrochloride], nitroglycerin (NTG, Beijing Yimin Pharmaceutical Co., Ltd., Beijing, China), okadaic acid, Rp isomer (Rp-8-Br-PET-cGMPS; Biolog Life Science Institute, Bremen, Germany), and U46619 (9,11-Dideoxy-11*α*,9*α*-epoxymethanoprostaglandin F2*α*).

LY294002, calyculin A, and okadaic acid were dissolved in DMSO (final concentration; <0.2%). Preliminary experiments showed that DMSO at the concentration used had no effect on contraction to U46619 and relaxation induced by nitroglycerin. The other drugs were prepared using distilled water.

### 2.2. Porcine Coronary Arteries Preparations

Fresh porcine hearts were collected from a local slaughterhouse. The left anterior descending coronary arteries were carefully dissected and cut into rings in ice-cold modified Krebs-Ringer bicarbonate buffer. The vessels were denuded of the endothelium mechanically by inserting the tips of a watchmaker's forceps into the lumen of the vessel and rolling the vessel back and forth on saline-saturated filter paper. Animal handling and study protocols were in accordance with US National Institutes of Health guidelines. They were reviewed and approved by Animal Care and Use Review Committees of Peking University Health Science Center [31].

### 2.3. Vessel Tension Study

Rings of porcine coronary arteries were repeatedly rinsed and suspended in organ chambers filled with 10 mL of the modified Krebs-Ringer bicarbonate solution maintained at 37 ± 0.1°C and aerated with 95% O_2_-5% CO_2_ (pH = 7.4). Two stirrups passed through the lumen of the vessel ring: one was anchored to the bottom of the organ chamber and the other was connected to a strain gauge. The isometric force was measured with ML785 PowerLab/8sp recording and Analysis System (ADInstruments Pty Ltd., Castle Hill, Australia) [32].

At the beginning of the experiment, each vessel was stretched to its optimal resting tension by stepwise stretching until the contraction of the vessel ring to 100 mM KCl reached a plateau. The optimal resting tension of porcine coronary arteries was ~2.5 g. Then one hour of equilibration was allowed. After equilibration certain inhibitors including ODQ, Rp-8-Br-PET-cGMPS, and LY294002 were added and remained throughout the experiment. In some experiments, NTG, isoproterenol, DETA NONOate, and/or 8-Br-cGMP were added 30 minutes before constriction. The effects of hypoxia (95% N_2_-5% CO_2_) were examined in vessels preconstricted with U46619 to a similar tension level. To eliminate the possible involvement of endogenous prostanoids and endothelium-derived nitric oxide, indomethacin (10^−5^ M, an inhibitor of cyclooxygenase) and nitro-L-arginine (10^−4^ M, an inhibitor of NO synthase) were administrated. All experiments were carried out in a parallel fashion.

### 2.4. Western Blot Study

Pretreatment of arterial rings: isolated porcine coronary arteries without endothelium were incubated in Krebs-Ringer bicarbonate buffer maintained at 37 ± 0.1°C and aerated with 95% O_2_-5% CO_2_ (pH = 7.4) in the presence of solvent, NTG, DETA NONOate, or 8-Br-cGMP. The inhibitors including ODQ, Rp-Br-PET-cGMPS, calyculin A, and okadaic acid were added at least 30 minutes before testing their effects.

Immunodetection: vessel rings were rapidly frozen with liquid nitrogen and homogenized in lysis buffer containing 34.67 mM SDS, 1 mM sodium orthovanadate, and 10 mM Tris base (pH 7.4). Tissue lysates each containing 20 *μ*g of soluble protein were subjected to SDS-PAGE and electrotransferred to polyvinylidene fluoride membrane. Nonspecific binding of antibody was blocked by washing with TBS buffer containing 5% milk for 1 hour at room temperature. After two times brief washing with TBS plus 0.1% Tween-20 (TBS-T), the blot was incubated with the first antibody of Akt (Cell Signaling Technology, MA, USA; 1 : 1000 dilution) or Akt-p (S473) (Cell Signaling Technology, MA, USA; 1 : 1000 dilution) overnight at 4°C. Afterwards, the blot was washed three times with TBS-T buffer and then incubated with the secondary antibody for 1 hour at room temperature followed by another 3 times of washing and then developed using the chemiluminescent detection method (Amersham ECL). Proteins of Akt or Akt-p were quantified by densitometry using a Gel Doc 2000 densitometer (BIO-RAD, CA, USA) and normalized to scanning signals of beta-actin (Calbiochem, CA, USA).

### 2.5. Data Analysis

Data are shown as means ± SEM. Student's *t*-test for unpaired observations was used to compare the mean values of two groups. Mean values of more than two groups were compared using one-way ANOVA test, with the Student-Newman-Keuls test for* post hoc* testing of multiple comparisons. Statistical significance was accepted when the *P* value (two tailed) was less than 0.05. In all experiments, *n* represents the number of animals.

## 3. Results

### 3.1. Hypoxia-Induced Sustained Vasoconstriction Was Prevented Specifically by NTG

**Figure 1:**
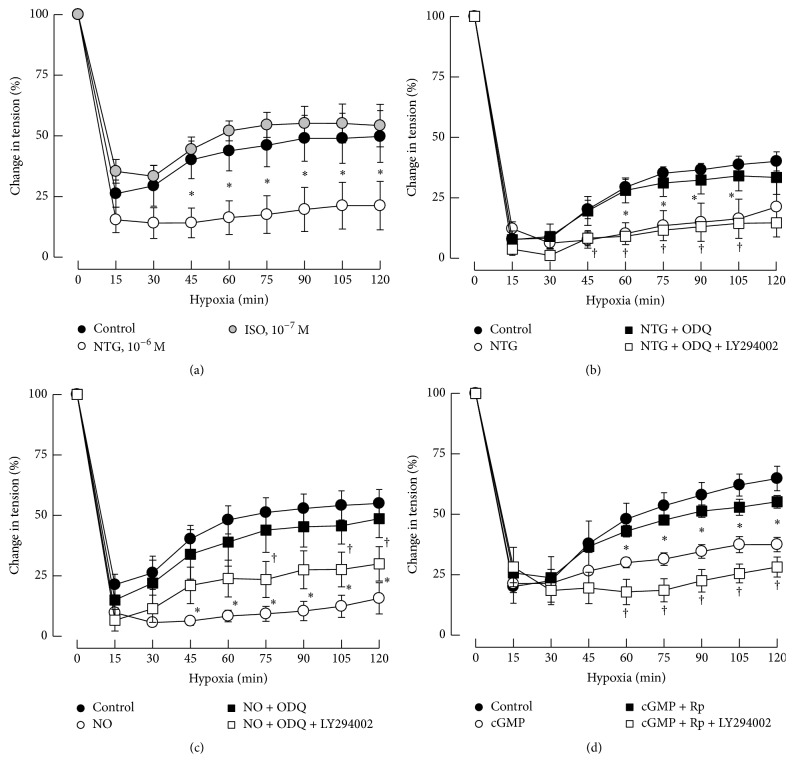
Effects of NTG, ISO, DETA NONOate, and cGMP on hypoxic vasoconstriction. The continuous contraction caused by hypoxia in porcine coronary arteries was prevented by NTG (10^−6^ M), but not by isoproterenol (ISO, 10^−7^ M; (a)). The prevention of the hypoxic vasoconstriction caused by NTG (10^−6^ M; (b)), DETA NONOate (NO, 10^−4^ M; (c)), and 8-Br-cGMP (cGMP, 10^−4 ^M; (d)) was recovered by ODQ (3 × 10^−5^ M, a specific inhibitor of sGC; (b) and (c)) and Rp-Br-PET-cGMPS (PKG-I; 3 × 10^−5^ M; (d)). The restored hypoxic contraction of porcine coronary arteries by ODQ or Rp-Br-PET-cGMPS was reversed by LY294002 (10^−5^ M, a specific inhibitor of PI3K; (b), (c), and (d)). Data are means ± SEM; *n* = 4–6 for each group. ^*∗*^significantly different between the control group and treatment group with NTG, DETA NONOate, or 8-Br-cGMP (*P* < 0.05); ^†^significantly different between the group with LY294002 and the group without LY294002 (*P* < 0.05).

Rings of porcine coronary arteries without endothelium were preconstricted with U46619 (3 × 10^−7^ M, a thromboxane A2 analog) before testing the response of hypoxia (95% N_2_-5% CO_2_). Hypoxia induced a rapid relaxation in the first 30 min, which was followed by a sustained contraction in porcine coronary arteries (Figure 1(a)). This sustained contraction of porcine coronary arteries was prevented by NTG (10^−6^ M; Figure 1(a)), but not by isoproterenol (ISO, 10^−7^ M; Figure 1(a)). Our preliminary experiment showed that isoproterenol (10^−7^ M) induced the same relaxation as NTG (10^−6^ M) in porcine coronary arteries preconstricted with U46619 (3 × 10^−7^ M, data not shown).

### 3.2. Involvement of NO/sGC/cGMP/PKG Pathway

The prevention of the hypoxic-induced vasoconstriction caused by NTG (10^−6^ M) could be recovered by ODQ (3 × 10^−5^ M), a specific inhibitor of sGC (Figure 1(b)). Pretreatment of coronary arteries with NTG downstream molecule, NO donor (DETA NONOate, 10^−5^ M), or cGMP analog (8-Br-cGMP, 10^−4^ M) also attenuated the vasoconstriction caused by continuous hypoxia. These effects were blocked by ODQ or the inhibitor of PKG (PKG-I, Rp-8-Br-PET-cGMPS, 3 × 10^−5 ^M). ODQ and PKG-I themselves had no effect on the hypoxic vasoconstriction (Figures 1(b), 1(c) and 1(d)).

### 3.3. Role of PI3K/Akt

The prevention of hypoxia-induced vasoconstriction of porcine coronary arteries caused by NTG, NO, or cGMP could be recovered by ODQ or PKG-I and the restored contractions were largely abolished by coincubation with LY294002, a specific inhibitor of PI3K (Figures 1(b), 1(c) and 1(d)).

### 3.4. Involvement of sGC in the Downregulation of Akt-p (S473) by NTG and NO

**Figure 2:**
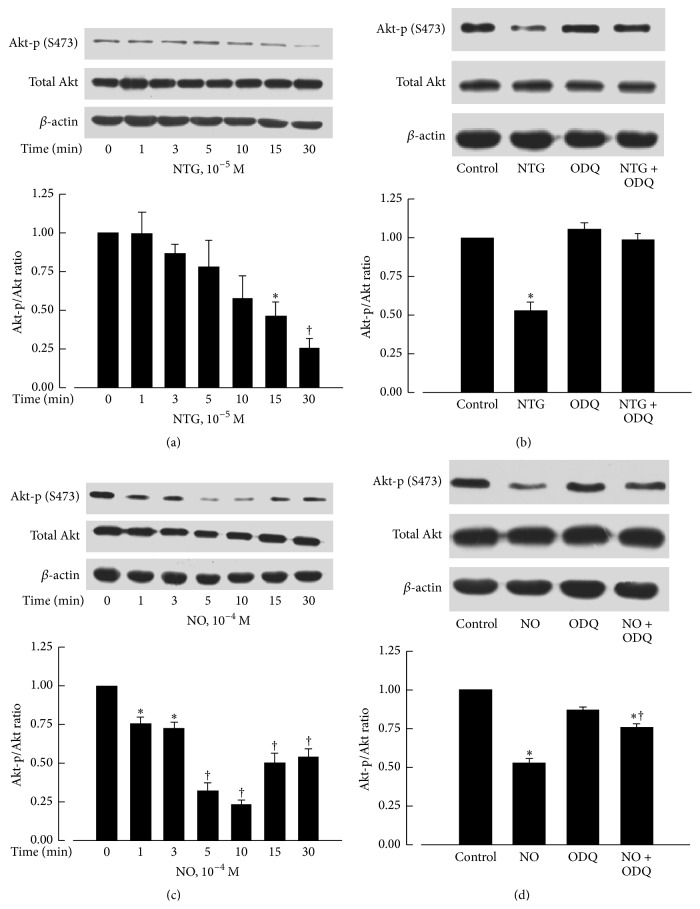
Effects of NTG and DETA NONOate on protein levels of Akt-p (S473) and total Akt. Akt-p (S473) was significantly decreased after NTG (10^−5^ M) treatment for 15 min (a) or DETA NONOate (NO, 10^−4^ M) treatment for 1 min (c), which could be recovered by ODQ (3 × 10^−5 ^M; (b) and (d)). The upper panels are western blots. The lower panels are the summaries of densitometric scanning of proteins expressed as ratio of Akt-p (S473) to total Akt. Data shown as means ± SEM; *n* = 6–8 for each group. ^*∗*^significantly different from the control group (*P* < 0.05); ^†^significantly different from the control group (*P* < 0.01, (a) and (c)); ^†^significantly different from the NO group (*P* < 0.05, (d)).

The protein levels of phosphorylated Akt at Ser-473 were significantly reduced by incubation with NTG (10^−5^ M) for 15 and 30 minutes (Figure 2(a)) or by incubation with DETA NONOate (10^−4^ M) for 1 to 30 minutes (Figure 2(c)). These effects were prevented by ODQ (3 × 10^−5^ M), a specific inhibitor of sGC (Figures 2(b) and 2(d)).

### 3.5. Involvement of PKG in the Downregulation of Akt-p (S473) by 8-Br-cGMP

**Figure 3:**
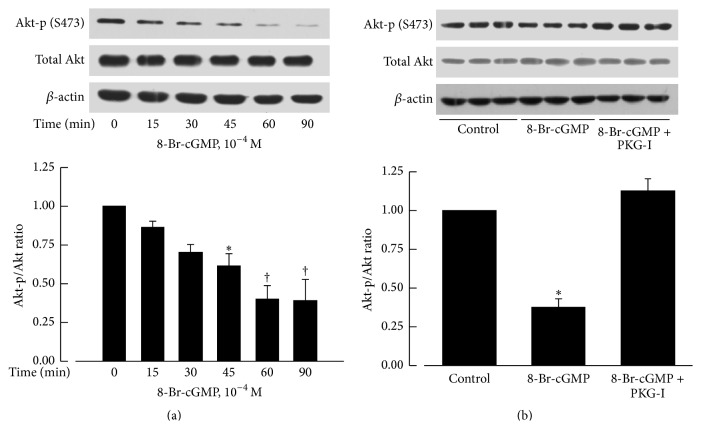
Effects of 8-Br-cGMP on protein levels of Akt-p (S473) and total Akt. Akt-p (S473) level was significantly reduced after 8-Br-cGMP (10^−4^ M) treatment for 45 min (a) and this effect could be prevented by Rp-8-Br-PET-cGMPS (PKG-I, 3 × 10^−5 ^M; (b)). The upper panels are western blots. The lower panels are the summaries of densitometric scanning of proteins expressed as ratio of Akt-p (S473) to total Akt. Data shown as means ± SEM; *n* = 6 for each group. ^*∗*^significantly different from the control group (*P* < 0.05); ^†^significantly different from the control group (*P* < 0.01).

The protein levels of phosphorylated Akt at Ser-473 were also significantly reduced by incubation with 8-Br-cGMP (10^−4^ M) for 45 to 90 minutes. The effect was blocked by Rp-8-Br-PET-cGMPS (3 × 10^−5^ M), a specific inhibitor of PKG (Figure 3).

### 3.6. Involvement of PP1 in the Downregulation of Akt-p (S473) by NTG and 8-Br-cGMP

**Figure 4:**
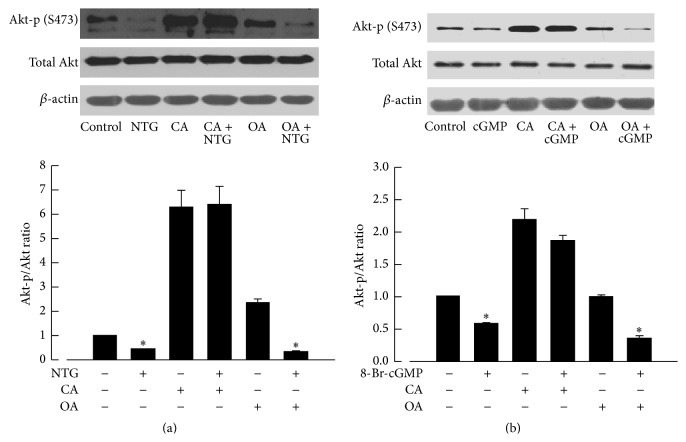
Effects of calyculin A and okadaic acid on the decrease in protein levels of Akt-p (S473) caused by NTG and 8-Br-cGMP. Akt-p (S473) was reduced when treated with NTG (10^−6^ M; (a)) for 15 min and 8-Br-cGMP (cGMP analog, 10^−4^ M; (b)) for 60 min. The reduction of Akt-p (S473) was prevented by calyculin A (CA, 10^−7^ M) but not by okadaic acid (OA, 10^−7^ M). The upper panels are western blots. The lower panels are the summaries of densitometric scanning of proteins expressed as ratio of Akt-p (S473) to total Akt. Data shown as means ± SEM; *n* = 6 for each group. ^*∗*^significantly different from the control group (*P* < 0.05).

Akt-p/Akt was decreased when treated with NTG (10^−6 ^M; Figure 4(a)) for 15 min and 8-Br-cGMP (cGMP analog, 10^−4 ^M; Figure 4(b)) for 60 min. Calyculin A (10^−7^ M, an inhibitor of PP1 and PP2A) but not okadaic acid (10^−7^ M, an inhibitor of PP2A) prevented the decrease in Akt-p (S473) caused by NTG and 8-Br-cGMP (Figure 4).

## 4. Discussion

Coronary artery spasm is a risk factor of acute ischemia heart disease such as angina pectoris and acute coronary syndrome [33, 34]. Endothelial dysfunction, hyperactivity of vascular smooth muscle cells, and other factors, including hypoxia, may be involved in the development of coronary vasospasm [2, 3, 34]. Recently, we found that prolonged hypoxia induced a transient initial contraction followed by a short term relaxation and a sustained contraction in porcine coronary arteries [10]. The first rapid hypoxic contraction is endothelium- or NO-dependent as reported by Vanhoutte and others [4, 9] while the second sustained contraction triggered by hypoxia is endothelium-independent [10]. Our study suggests that when oxygen content in the blood is decreased under certain disease conditions such as sleep apnea, high altitude sickness, and chronic obstructive pulmonary disease, the sustained hypoxic vasoconstriction may contribute to the development of spasm in coronary artery.

Both NTG and isoproterenol are vasodilators used in the treatment of cardiovascular disease. It is well known that vasodilatation caused by NTG is predominantly mediated by sGC/cGMP signaling pathway [28, 29] and the relaxation caused by isoproterenol involves adenylyl cyclase/cAMP signaling [35]. In our study, preincubation of nitroglycerin prevented the sustained vasoconstriction induced by hypoxia. By contrast, treatment of coronary arteries with isoproterenol had little effect. These results suggest that the endothelium-independent hypoxia-induced contraction is prevented specifically by NTG.

NTG causes vasodilatation via the intracellular conversion to NO or a NO related intermediate, which elevates cGMP by activating sGC [28, 29]. We found that the endothelium-independent hypoxic contraction was also prevented by NO. The suppression of hypoxic vasoconstriction by NTG or NO was recovered by ODQ, a specific inhibitor of sGC, suggesting that cGMP is involved in the effect of NTG and NO on hypoxia-induced vasoconstriction. PKG is one of the primary targets of cGMP. In our study the cGMP analog decreased the contraction induced by prolonged hypoxia in a manner sensitive to the inhibition of PKG by Rp-8-Br-PET-cGMPS. Thus, the activation of NO/sGC/cGMP/PKG pathway is involved.

Our previous work demonstrates that the activation of PI3K/Akt by hypoxia plays an important role in hypoxia-induced vasoconstriction [10]. This hypoxia-induced Akt phosphorylation at Ser-473 and vasoconstriction could be abolished by LY294002, an inhibitor of PI3K [10]. The present study shows that inhibition of PI3K/Akt pathway by LY294002 inhibited the effect of ODQ and Rp-8-Br-PET-cGMPS on the suppression of hypoxic vasoconstriction caused by NTG, NO, and 8-Br-cGMP. Hence, it appears that NTG prevents the hypoxia-induced coronary vasoconstriction mainly by inhibition of PI3K/Akt pathway.

Akt is a serine/threonine protein kinase involved in various cellular processes including the modulation of vascular smooth muscle responses [17]. In the present study the treatments of porcine coronary arteries with NTG, NO, or 8-Br-cGMP reduced the Akt phosphorylation at Ser-473, which was blocked by the inhibitors of sGC and PKG. It suggests that PKG activated by cGMP exerts an inhibitory effect on Akt by decreasing the phosphorylation of Akt at Ser-473, which is in line with our observation obtained in porcine pulmonary arteries [30].

PKG exerts its effects by phosphorylating the target proteins, including calcium activated potassium channels (BK channels) [36], IP_3_R-associated PKG substrate [37], RhoA [38], and myosin phosphatase targeting subunit (MYPT1) [39]. The present study showed that Akt was dephosphorylated rather than phosphorylated by PKG, indicating that PKG might act on Akt indirectly. Indeed, some studies show that dephosphorylation of Akt is mediated by protein phosphatase 1 (PP1) or protein phosphatase 2A (PP2A) [40]. To determine whether PP1 and PP2A played a role in the dephosphorylation of Akt caused by PKG, the effects of calyculin A (CA), a nonselective inhibitor of PP1 and PP2A, and okadaic acid (OA), a selective inhibitor of PP2A, were studied [41, 42]. We found that the reduced phosphorylation of Akt induced by NTG and cGMP was reversed by CA but not by OA. Therefore, PKG may reduce the phosphorylation of Akt at Ser-473 mostly through PP1.

We herein demonstrate that pretreatment of NTG can prevent endothelium-independent vasoconstriction caused by prolonged hypoxia in porcine coronary arteries. The underlying mechanism may involve the activation of PKG induced by NTG, NO, or cGMP, which subsequently attenuates the phosphorylation of Akt at Ser-473 through PP1 in porcine coronary arteries, resulting in the inhibition of PI3K/Akt signaling pathway.
